# Peripheral blood natural killer cell percentages in granulomatosis with polyangiitis correlate with disease inactivity and stage

**DOI:** 10.1186/s13075-015-0851-7

**Published:** 2015-11-21

**Authors:** Wolfgang Merkt, Prisca Sturm, Felix Lasitschka, Theresa Tretter, Carsten Watzl, Daniel Saure, Michael Hundemer, Vedat Schwenger, Norbert Blank, Hanns-Martin Lorenz, Adelheid Cerwenka

**Affiliations:** Department of Hematology, Oncology and Rheumatology, Internal Medicine V, University Hospital of Heidelberg, Im Neuenheimer Feld 410, 69120 Heidelberg, Germany; Innate Immunity Group, German Cancer Research Center, Heidelberg, Germany; Department of Pathology, University Hospital Heidelberg, Heidelberg, Germany; Leibniz Research Center for Working Environment and Human Factors, Dortmund, Germany; Institute of Medical Biometry and Informatics, University of Heidelberg, Heidelberg, Germany; Department of Nephrology, University Hospital of Heidelberg, Heidelberg, Germany

**Keywords:** Granulomatosis with polyangiitis, Natural killer cells, Vasculitis, Granuloma

## Abstract

**Introduction:**

The role of CD3−CD56+ natural killer (NK) cells in granulomatosis with polyangiitis (GPA) is poorly understood. Recently, it has been shown that peripheral blood NK cells can kill renal microvascular endothelial cells, suggesting a pathogenic role of NK cells in this disease. So far, subset distribution, phenotype, and function of peripheral blood NK cells in relation to GPA disease activity have not been elucidated. Moreover, it is not known whether NK cells infiltrate GPA tissue lesions.

**Methods:**

Paraffin sections of GPA granulomas and controls were stained with anti-CD56 and anti-CD3 antibodies. Peripheral blood lymphocyte subsets were analyzed by flow cytometry. NK cell degranulation was analyzed using cocultures of patient PBMCs with target cells and surface expression of CD107a. Clinical data were extracted from medical records. Statistical analysis was performed in an exploratory way.

**Results:**

CD56+ cells were not detectable in active granulomatous GPA lesions but were found frequently in granulomas from tuberculosis and sarcoidosis patients. In GPA, the proportion of NK cells among peripheral blood lymphocytes correlated negatively with the Birmingham Vasculitis Activity Score (BVAS) (n = 28). Accordingly, NK cell percentages correlated positively with the duration of remission (n = 28) and were significantly higher in inactive GPA (BVAS = 0, n = 17) than in active GPA, healthy controls (n = 29), and inactive control diseases (n = 12). The highest NK cell percentages were found in patients with long-term remission and tapered immunosuppressive therapy. NK cell percentages >18.5 % of peripheral blood lymphocytes (n = 12/28) determined GPA inactivity with a specificity of 100 %. The differentiation into CD56^dim^ and CD56^bright^ NK cell subsets was unchanged in GPA (n = 28), irrespective of disease activity. Similar surface expression of the activating NK cell-receptors (NKp30, NKp46, and NKG2D) was determined. Like in healthy controls, GPA NK cells degranulated in the presence of NK cell receptor ligand bearing epithelial and lymphatic target cells.

**Conclusions:**

NK cells were not detectable in GPA granulomas. Peripheral blood NK cell percentages positively correlate with the suppression of GPA activity and could serve as a biomarker for GPA activity. Peripheral blood NK cells in GPA patients are mature NK cells with preserved immune recognition.

## Introduction

Granulomatosis with polyangiitis (GPA) is a systemic inflammatory disease. European League Against Rheumatism (EULAR) treatment recommendations published in 2009 [1] propose an induction therapy consisting of cyclophosphamide and high-dose steroids followed by a maintenance therapy with disease-modifying antirheumatic drugs (DMARDs) and low-dose steroids. Recent studies showed high efficacy of the anti-CD20 antibody rituximab in induction [2–4] and maintenance therapies [5, 6]. To date, it is unclear how long maintenance therapy should be continued.

The Birmingham Vasculitis Activity Score (BVAS) is a standard activity score used in studies of GPA. The presence of anti-proteinase 3 antibodies (PR3) is specific for GPA. However, consideration of PR3 serum levels as an activity marker has been discussed controversially [7]. So far, no laboratory test can reliably be used as an activity biomarker [8].

Granulomas and vasculitis define GPA histologically. GPA granulomas consist of granulocytes surrounded by T and B cells [9, 10]. Granulocytes express the key antigen proteinase 3. So far, mainly granulocytes, macrophages, fibroblasts, T cells, and B cells have been studied in GPA [10–15]. Knowledge about an implication of natural killer (NK) cells in GPA is very limited. Tognarelli et al. [16] recently reported that NK cells from patients with GPA can directly kill renal microvascular endothelial cells (MECs) ex vivo. They concluded that NK cells may participate in vasculitis of the kidney. However, they studied NK cell frequency, subset distribution, phenotype, and function of peripheral blood NK cells in relatively few patients and not in relation to GPA disease activity. Importantly, whether NK cells can be found in GPA tissue lesions is currently unknown.

Among peripheral blood lymphocytes (PBLs), NK cells make up a considerable proportion (5–15 %). They provide a first-line defense against intracellular pathogens and tumors [17, 18]. Two major subsets are well established: CD56bright NK cells are dominant in secondary lymphatic tissues and presumably parental cells of CD56dim NK cells. CD56dim NK cells are mature and more cytotoxic cells and make up >90 % of peripheral blood NK cells. NK cell effector mechanisms are cytotoxicity and secretion of inflammatory cytokines and chemokines. The surface expression of the NK cell degranulation marker CD107a is strongly associated with NK cell cytotoxicity [19]. NK cell immune recognition and activity are balanced by activating and inhibiting receptors [20], but they can be modulated on several levels [21]. NK cells are regarded as a link between innate and adaptive immunity [22]. Importantly, adaptive immunity can be modulated by NK cells at different stages [23], such as by secretion of interleukin (IL)-10 [24] and killing of other immune cells, including activated CD4^+^ T cells and B cells [25–27].

## Material and methods

### Patient consent and ethical approval

Informed consent was obtained from the patients before study initiation. The ethics committee of the University of Heidelberg approved this study.

### Histology

Paraffin-embedded sections from 13 GPA granulomas (12 in lungs, 1 in kidney) of 10 different patients with GPA and each 5 granulomas from patients with tuberculosis and sarcoidosis were processed. The following primary antibodies were used: rabbit anti-human monoclonal antibody (mAb) CD3 (clone SP7, immunoglobulin G (IgG), 1:200; Thermo Fisher Scientific, Fremont, CA, USA) and mouse anti-human CD56 mAb (clone 1B6, IgG1, 1:10; Leica Biosystems, Newcastle upon Tyne, UK). Isotype- and concentration-matched mouse and rabbit control mAb (Dianova, Hamburg, Germany) served as negative controls. Immunoenzyme staining was performed on 2-μm paraffin-embedded sections of formalin-fixed tissue using automated staining with the DAKO autostainer according to the manufacturer’s instructions (Dako, Glostrup, Denmark). Antigen retrieval for CD3 was achieved by steam-cooking the slides in 1 mM ethylenediaminetetraacetic acid buffer (pH 9; Dako) and for CD56 in 10 mM citrate buffer (pH 6.1; Dako) for 30 minutes. 3,3′-Diaminobenzidine was used as the substrate.

### Patients and controls

Heparinized blood was drawn from 30 patients with GPA. One patient was excluded because of concomitant B-cell malignancy, and another one was excluded because the diagnosis of GPA could not be retraced sufficiently. The 28 analyzed patients (Table 1) did not have significant comorbidities. Of note, in three patients with NK cell frequencies over 40 %, no common pathology apart from GPA was found in the available patient records. One female patient with 57.4 % NK cells had been in remission for almost 5 years and had received trastuzumab (HER2 antibody) for treatment of breast cancer. It cannot be excluded that trastuzumab was in part responsible for this high NK cell proportion. Exclusion of this patient from the analysis did not relevantly alter the statistical significance data presented in this study.

**Table 1:** Patient characteristics

Characteristics	Data
Patients with GPA, n	28
Male/female, n	16/12
Age, yr, mean ± SD (range)	61.8 ± 16.6 (30–81)
Patients with active GPA (BVAS >0), n (%)	11/28 (39 %)
BVAS (of active GPA), mean ± SD	6.8 ± 7.5
Patients with inactive GPA (BVAS = 0), n (%)	17/28 (61 %)
Duration of remission, yr (of inactive GPA), median ± SD	4.08 ± 2.3
Organ system involvement, n (%)	
ENT	23/28 (82 %)
Lung	15/28 (54 %)
Kidney	18/28 (64 %)
Skin	8/28 (29 %)
Joints	12/28 (43 %)
NS	8/28 (29 %)
Localized GPA (upper airways and ENT organs only), n (%)	3/28 (11 %)
Generalized GPA, n (%)	25/28 (89 %)
Number of involved organ systems, mean ± SD	4.3 ± 1.2
Number of relapses, mean ± SD (range)	1.1 ± 1.4 (0–5)
Length of time since disease onset, yr, mean ± SD	9.4 ± 7.9
c-ANCA, n (%)	
Positive	22/28 (79 %)
Negative	3/28 (11 %)
Not determinable	3/28 (11 %)

*c-ANCA* cytoplasmic antineutrophil cytoplasmic antibodies, *GPA* granulomatosis with polyangiitis, *ENT* ear, nose, and throat, *NS* nervous system, *PR3* anti-proteinase 3 antibody, *SD* standard deviation

A total of 29 volunteers (15 men, 14 women; median age 49.5 years) without systemic inflammatory diseases or other active illness served as healthy controls (HCs). Twelve patients with inactive systemic inflammatory diseases other than GPA represented a group constituting inactive control diseases (CDs): systemic lupus erythematosus (n = 4), panarteritis nodosa (n = 1), overlap connective tissue disease (n = 1), chronic inflammatory bowel disease (n = 1), CREST (calcinosis, Raynaud’s phenomenon, esophageal dysmotility, sclerodactyly, and telangiectasia) syndrome (n = 1), primary Sjögren’s syndrome (n = 1), polymyalgia rheumatica (n = 1), and giant cell arteritis (n = 2).

### Definitions and analysis of medical records

BVAS was determined at the time of blood donation (i.e., the day of inclusion in the study). Inactive and active GPA were defined by BVASs of 0 and ≥1, respectively. As BVAS was not determined in routine clinical practice, the following terminology was used to retrospectively analyze disease courses. *Relapses* were defined by major disease activity necessarily resulting in reinduction therapy. *Disease activity* included initial disease flares and relapses as well as every situation with GPA activity that did not result in (re-)induction therapy but met at least one of the following criteria: (1) new or augmented organ involvement, (2) entry activity or relapse in medical report, and (3) increased immunosuppressive therapy. *Duration of remission* was defined by the length of time since last disease activity.

### Flow cytometry

Peripheral blood mononuclear cells (PBMCs) were isolated using Ficoll-Paque density gradient medium (GE Healthcare Life Sciences, Uppsala, Sweden) and incubated for 30 minutes on ice with a mixture of antibodies (fluorescein isothiocyanate anti-CD3; phycoerythrin/Cy7 anti-CD56 in every experiment; and additionally in some experiments, allophycocyanin anti-CD19; all from BioLegend, San Diego, CA, USA) and 7-aminoactinomycin D (7-AAD; BD Biosciences, San Jose, CA, USA). After being washed, PBMCs were resuspended in a fixation solution and immediately analyzed by flow cytometry. Lymphocyte subsets from 9 of the 14 CD patients were analyzed according to our clinical laboratory routine using a standard antibody kit from Beckman Coulter (Brea, CA, USA).

### Degranulation (CD107a) assays

PBMCs were isolated as described, frozen the same day, thawed the day before the experiment, and cultured overnight. PBMCs (10^5^) were cocultured for 4 h (37 °C, 5 % CO_2_) with target cells in a 1:1 ratio. Cocultures were performed in duplicates in 200 μl of RPMI per well on a 96-well plate. Anti-CD107a mAb (BioLegend) was added at the beginning of the coculture in combination with 0.25 μl of Golgi-Stop (BD Biosciences). After two washing steps, PBMCs and target cells were incubated with antibodies for cell surface staining and analyzed by flow cytometry as described above. Additive effect on degranulation is defined by the percentage of NK cells expressing the degranulation marker CD107a after incubation with target cells minus the percentage of NK cells expressing the degranulation marker CD107a after incubation without target cells.

As target cells, major histocompatibility complex class I–positive BxPC-3 (pancreatic carcinoma) cells and JE6-1 (leukemic Jurkat) cells were used [retrovirally transfected with pMXneo (vector control, VC) and pMXneo-CD8L-Myc tag-B7-H6, respectively, and cultured as previously described] [28]. B7-H6 expression was controlled using an anti-B7-H6 antibody [28].

### Statistical analysis

The statistical analysis was performed in an exploratory way. The *p* values have to be interpreted descriptively. A normal distribution of NK cell percentages and counts was not assumed; therefore, non-parametric statistical tests were used. The Kruskal-Wallis test and Dunn’s posttest were used for multiple comparisons. The Mann-Whitney *U* test was used to compare two patient groups. Spearman’s test was used to investigate correlations. Receiver operating characteristic (ROC) curves were created to investigate the usefulness of parameters as a test to distinguish between two groups. On the basis of ROC curves, suitable threshold values delivering the best test performance were determined. A paired *t* test was used to compare NK cells of the same donor after coculture with differently transfected target cells. All tests were performed with a significance level of 5 % (confidence interval 95 %).

## Results

### CD56^+^ cells were not detectable in GPA but were present in granulomas of classical granulomatous diseases

**Fig. 1:**
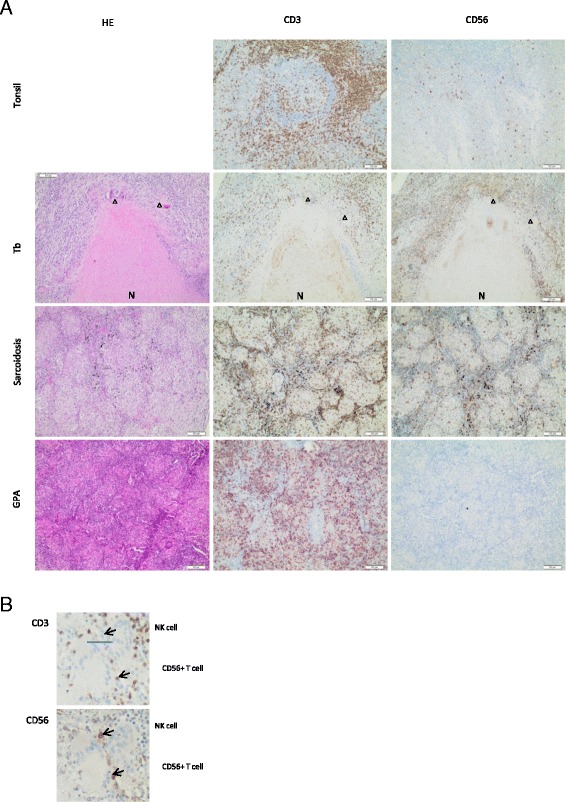
CD56^+^ cells are not detectable in granulomatosis with polyangiitis (GPA) but are present in granulomas of classical granulomatous diseases. Patient material was derived from and processed in the Heidelberg tissue bank for inflammatory diseases. Sequential paraffin-embedded sections were stained with hematoxylin and eosin (HE) (*left column*), anti-CD3 antibody (*middle column*), and anti-CD56 antibody (*right column*). **a** Normal tonsil tissue served as a positive control for CD3 and CD56 staining (*top row*). Representative tissue sections of granulomas from tuberculosis (Tb) (n = 5; *second row*), sarcoidosis (n = 5; *third row*), and GPA (n = 13; *bottom row*) patients. Healthy lung tissue at the border of GPA granulomas contained scattered CD56^+^ cells (not shown). *Arrowheads* point to giant cells. *N* necrosis. Measurement bars indicate 100µm. **b** Enlarged cutout from the sequential Tb sections shown in (a). *Upper arrows*, CD3^−^CD56^+^ natural killer (NK) cells; *lower arrows*, CD3^+^CD56^+^ T cells

Thirteen histological sections of GPA granulomas were stained for CD3 and CD56 (Fig. 1a). All GPA granulomas were completely negative for CD56 staining. However, scattered CD56^+^ cells were found in normal lung tissue at section borders (not shown). The non-detectable levels of CD56 were specific for GPA, as abundant CD56^+^ cells were found in each of five tuberculosis and sarcoidosis granulomas (Fig. 1a). Presumably, at least a few CD56^+^ cells in tuberculosis and sarcoidosis granulomas represented NK cells because, despite the difficult identification of CD3-CD56^+^ cells in sequential sections due to massive CD3^+^ infiltrates, some CD3-CD56^+^ cells could be identified (Fig. 1b).

### NK cell percentages in PBLs negatively correlate with GPA activity and are increased in inactive GPA

**Fig. 2:**
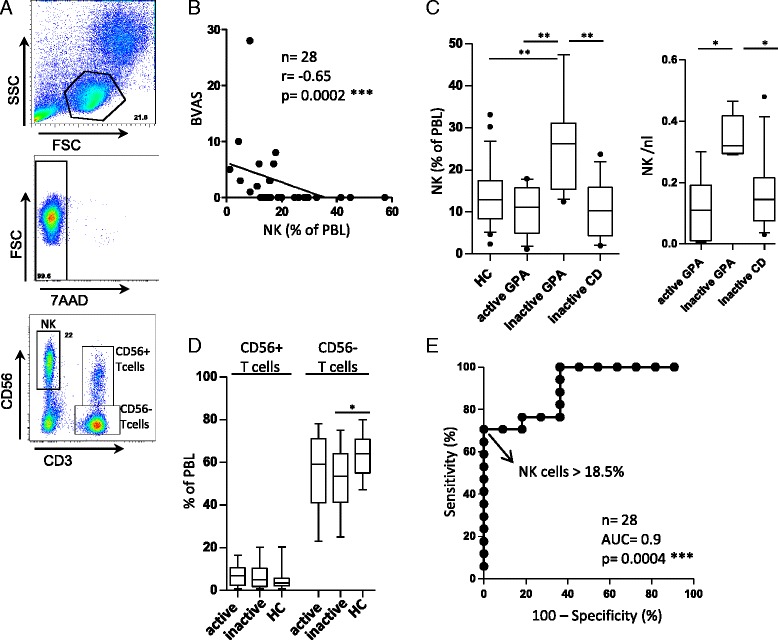
Natural killer (NK) cell proportions negatively correlate with granulomatosis with polyangiitis (GPA) activity and are increased in inactive GPA. Peripheral blood mononuclear cells from patients and healthy controls (HCs) were isolated using Ficoll-Paque density gradient medium and analyzed by flow cytometry. **a** Gating strategy. Peripheral blood lymphocytes (PBLs) were determined using forward and sideward scatter (FSC and SSC, respectively). Cell viability was confirmed by negative 7-aminoactinomycin (7-AAD) staining in each experiment. NK cells were determined by costaining of CD3 (negative) and CD56 (positive). **b** Scatterplot showing the relationship between NK cell proportions and Birmingham Vasculitis Activity Score (BVAS). Note that in one case, two patients are represented by a single dot; both had a BVAS of 3 and NK cell proportions of 15.6 % and 15.8 %, respectively (n = 28). Spearman’s correlation coefficient (*r*) and statistical significance were as indicated on the figure. **c** and **d** Box-and-whisker plots. Whiskers represent 10th–90th percentile range, statistical significance is as indicated. *Left graph* in (**c**) depicts NK cell proportions of PBLs from HCs (n = 29), patients with active GPA (n = 11), patients with inactive GPA (n = 17), and patients with inactive systemic inflammatory control diseases (CD; n = 12). *Right graph* in (**c**) illustrates NK cell counts in PBLs from patients with active GPA (n = 6), patients with inactive GPA (n = 5), and patients with inactive CD (n = 12). Statistical significance was determined by Kruskal-Wallis test (*p* = 0.0002 for relative and *p* = 0.014 for absolute NK cell numbers) and Dunn’s multiple comparison posttests (asterisks) as shown in the graphs. **d** T-cell proportions of patients with active (n = 11), inactive (n = 17) GPA and of HCs. Statistical analysis using the Kruskal-Wallis test revealed that CD56^−^ but not CD56^+^ T-cell proportions were significantly different (*p* = 0.04). Dunn’s multiple comparison posttests were positive where indicated by asterisks. **e** Receiver operating characteristic curve showing sensitivity and specificity of different NK cell proportions to distinguish inactive from active GPA. NK cells >18.5 % of PBLs determined GPA inactivity with a specificity of 100 % and a sensitivity of 71 %. *AUC* area under the curve

NK cells of patients and HCs were defined as living (7-AAD–negative) CD3^−^CD56^+^ cells (Fig. 2a) and showed comparable expression of the NK-specific marker NK cell p46-related protein (NKp46; not shown). In addition, CD3^−^CD56^+^ cells were consistently CD19^−^ (not shown).

A correlation study was performed after pooling of all patients (n = 28) independently of disease activity. NK cell proportions correlated negatively with BVAS (*r* = −0.65, *p* = 0.0002) (Fig. 2b). In a subset of patients, absolute lymphocyte counts were determined for diagnostic purposes, which enabled us to calculate absolute NK cell counts. Absolute NK cell counts confirmed the negative correlation with BVAS (n = 11, *r* = −0.79, *p* = 0.0047) (not shown).

Patients with inactive GPA (n = 17) had significantly greater NK cell proportions in PBLs than did patients with active GPA (n = 11) and HCs (n = 29) (Fig. 2c, *left graph*). This increase was not a generalizable feature of immune disease in remission, as shown by comparison with inactive CD (n = 12) (Fig. 2c). Similar to NK cell proportions, absolute NK cell counts were significantly increased in inactive GPA (n = 5) compared with active GPA (n = 6) and inactive CD (n = 12) (Fig. 2c, *right graph*).

As HCs were slightly younger than patients with GPA (Table 1), we examined a possible influence of age on NK cell proportions. Patients with active GPA and patients with inactive GPA patients differed slightly in mean age (mean age 54 and 65 years, respectively). However, there was no statistically significant correlation between age and NK cell proportions in patients with GPA (Spearman’s *r* = 0.26, *p* = 0,187; not shown).

To exclude increased expression of CD56 on cell types that are not NK cells, we also determined CD56^+^ T-cell proportions. CD56^+^ T-cell proportions were similar in active (n = 11) and inactive (n = 17) GPA, as well as in HCs (n = 29) (Fig. 2d). CD56^−^ but not CD56^+^ T-cell proportions were significantly different (*p* = 0.04 by Kruskal-Wallis test). Compared with HCs, patients with inactive GPA showed statistically significantly lesser CD56^−^ T-cell proportions (Fig. 2d).

Next, we evaluated the power of NK cell proportions to determine GPA inactivity. A ROC curve was created (Fig. 2e) showing the sensitivity and specificity of different NK cell proportions to distinguish inactive from active GPA. NK cells >18.5 % of PBLs determined inactivity with a specificity of 100 % and a sensitivity of 71 %, resulting in a positive likelihood ratio >72. NK cells >18.5 % of PBLs were detected in 12 of 28 patients. The area under the curve was 0.9.

Together, these data demonstrate that NK cell proportions negatively correlated with GPA activity and were significantly increased in inactive GPA. A positive likelihood ratio >8 indicates that NK cell percentages can be used as an (in)activity biomarker [29].

### NK cell proportions correlate with duration of remission

**Fig. 3:**
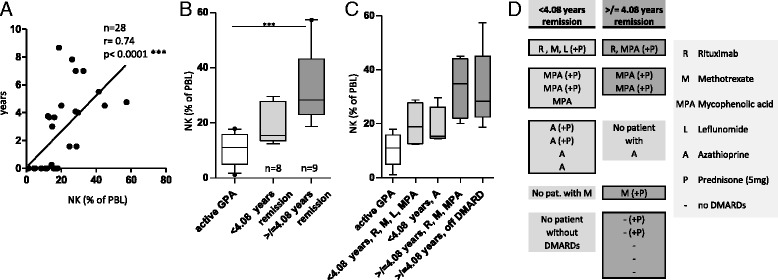
Natural killer (NK) cell proportions correlate with duration of remission. NK cell proportions of peripheral blood lymphocytes (PBLs) were determined by flow cytometry as described in Fig. 2. **a** Scatterplot showing the relationship between NK cell proportion and the duration of remission (in years). Spearman’s correlation coefficient (*r*) and statistical significance are as indicated above the graph. **b** Box-and-whisker plot showing NK cell proportions of inactive patients with GPA with <4.08 years of remission (n = 8) and ≥4.08 years of remission (n = 9). Statistical significance was determined by Kruskal-Wallis test (*p* = 0.0003) and Dunn’s multiple comparisons posttests (asterisks indicate statistical significance). **c** Reconfiguration of scatterplot shown in (b) after subgrouping according to disease-modifying antirheumatic drug (DMARD) therapy. **d** Overview of immunosuppressive maintenance/DMARD therapy. *Left column*, patients with <4.08 years of remission. *Right column*, patients with ≥4.08 years of remission. Each row of each column represents a single patient

NK cell proportions correlated with the duration of remission (n = 28, *r* = 0.74, *p* < 0.0001) (Fig. 3a). Likewise, absolute NK cell counts correlated with the duration of remission (n = 11, *r* = 0.69, *p* = 0.0198) (data not shown).

Principally, shifting of lymphocyte populations might be caused by medication. To further address the possible impact of disease stage and therapy, patients with inactive GPA were divided into two groups according to the median duration of remission (4.08 years) (Fig. 3b). Kruskal-Wallis analysis revealed significantly different NK cell proportions between patients with active GPA, patients with shorter remission, and patients with longer remission (Fig. 3b).

During clinical remission, maintenance therapy is usually tapered gradually. In our patient cohort, all patients with shorter remission (n = 8/8) received continued DMARD therapy (Fig. 3c, d). However, only 44 % of patients with longer remission (n = 4/9) received continued DMARD therapy. No different NK cell proportions were detectable after subgrouping according to DMARD type (Fig. 3c, d). Therefore, increased NK cell proportions were associated with both long-term remission and tapered maintenance therapy.

B-cell depletion by rituximab might increase remaining lymphocyte proportions. However, rituximab was infused in a minority of our patients (n = 5/28). The statistically significant difference between NK cell proportions from patients with active GPA and those with inactive GPA remained unchanged after elimination of these patients (not shown).

In investigating whether steroids influence NK cell numbers, we found a statistically significant but weak negative correlation between steroid dosage and NK cell proportions (*r* = −0.43, *p* = 0.03) (not shown). Steroid dosages in patients with active GPA were significantly higher than in those with inactive GPA (*p* = 0.04). All patients with inactive GPA received either low-dose steroids (equivalent of 5 mg of prednisone) or no steroids at all. Steroid dosages in inactive CD were similar to inactive GPA and equally significantly lower than in active GPA (*p* = 0.01).

Thus, we conclude that NK cell proportions correlate with duration of remission. The highest NK cell proportions were found in patients with long-term remission and tapered maintenance therapy. Rituximab, DMARDs, and (withdrawal of) steroids do not contribute to this increase in NK cells.

### The majority of peripheral blood NK cells in GPA and HC are mature CD56^dim^ NK cells

**Fig. 4:**
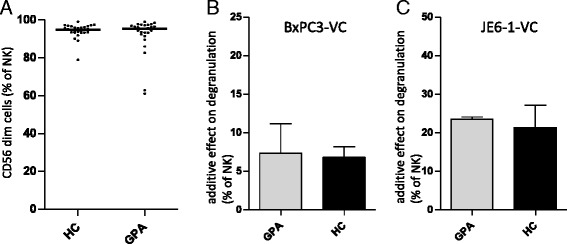
Natural killer (NK) cells in granulomatosis with polyangiitis (GPA) are mature cells with preserved competence for immune recognition. Peripheral blood mononuclear cells (PBMCs) were isolated and analyzed by flow cytometry as described in Fig. 2. **a** Dot blots showing the percentages of CD56^dim^ NK cells from healthy control subjects (HCs; n = 29) and patients with GPA (n = 28). **b**, **c** Bar graphs showing the additive effect on degranulation of NK cells after 4-h incubation of PBMCs with target cell lines. For further details of the experimental setup, see Material and methods section. **b** The additive effect on degranulation of NK cells from patients with GPA (n = 5; 2 active, 3 inactive) and HCs (n = 7) cultured with the epithelial cell line BxPC-3 transfected with a vector control (BxPC3-VC). **c** The additive effect on degranulation of NK cells from patients with GPA (n = 2; 1 active, 1 inactive) and HCs (n = 3) cultured with the JE6-1 (leukemic Jurkat) lymphatic cell line transfected with a vector control (JE6-1-VC). The additive effect on degranulation is defined by the percentage NK cells expressing the degranulation marker CD107a after incubation with target cells minus the percentage of NK cells expressing the degranulation marker CD107a after incubation without target cells. Statistical analysis was performed using the Mann-Whitney *U* test (not significant)

Concordant with the literature, most HC peripheral blood NK cells in our study corresponded to the CD56^dim^ subset (Fig. 4a). This was unchanged in samples of patients with GPA (Fig. 4a). The NK cell subset distribution was similar between active and inactive GPA (not shown).

NK cells from patients with GPA and HCs degranulated to a comparable degree in the presence of target cells from an epithelial or lymphatic origin (Fig. 4b, c).

**Fig. 5:**
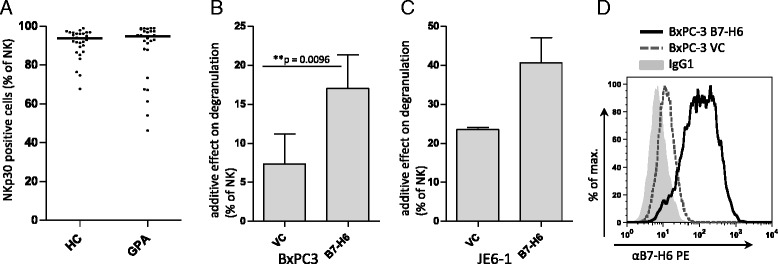
Natural killer (NK) cells from patients with granulomatosis with polyangiitis (GPA) can be activated via the NK cell p30-related protein (NKp30) pathway. Peripheral blood mononuclear cells (PBMCs) were isolated and analyzed by flow cytometry as described in Fig. 2. **a** Dot blots showing the percentages of NKp30^+^ NK cells from healthy control subjects (HCs; n = 29) and patients with GPA (n = 28). **b** and **c** Bar graphs depicting the additive effect on degranulation of NK cells after 4-h incubation of PBMCs of patients with GPA with target cell lines. The additive effect on degranulation of NK cells from PBMCs cultured with the vector controls (VCs) shown in Fig. 4b and c is contrasted with the additive effect on degranulation after incubation with (**b**) BxPC-3 (n = 5; 2 active, 3 inactive) and (**c**) JE6-1 (n = 2; 1 active, 1 inactive) overexpressing NKp30 ligand B7-H6, respectively. The additive effect on degranulation is defined by the percentage of NK cells expressing the degranulation marker CD107a after incubation with target cells minus the percentage of NK cells expressing the degranulation marker CD107a after incubation without target cells. *Statistical significance was determined using a paired *t* test. **d** Representative histogram showing the surface expression of B7-H6 on BxPC-3 transduced with B7-H6 or the VC. B7-H6 expression on transduced JE6-1 was comparable to that on BxPC-3. *IgG1* immunoglobulin G1 isotype control

To further investigate general NK cell functionality, we selected as an example the NK cell p30-related protein (NKp30) pathway. NKp30 is an important activating NK cell receptor with a known ligand, B7-H6. In the HC group, a median of 94 % of NK cells expressed NKp30 (Fig. 5a). This was unchanged in GPA (median 95 %). The percentage of NKp30^+^ NK cells was not different between active and inactive GPA (not shown). Surface overexpression of NKp30 ligand B7-H6 on target cells (Fig. 5d) significantly increased the degranulation of both HC (not shown) and GPA NK cells (Fig. 5b, c).

Finally, the proportion of NKp46^+^ NK cells in HC and GPA was identical (not shown). Almost all NK cells from HC and patients with GPA expressed NK group 2, member D (NKG2D; not shown).

These data demonstrate that NK cells in GPA are mature cells with preserved competence for target cell recognition.

## Discussion

Principally, NK cells could both promote and limit autoimmune inflammation [23, 30–32]. Tognarelli et al. implicated NK cells in the pathogenesis of GPA for the first time [16]. However, the presence of NK cells in GPA tissue lesions has not yet been investigated, and blood NK cells have not yet been correlated to disease activity. Correlation to disease activity is a pivotal step toward increasing understanding of the function of NK cells in systemic inflammatory diseases [30].

The first major result of our study is that CD56^+^ cells were not detectable in GPA granulomas, but in granulomas from sarcoidosis and tuberculosis. The interpretation of this finding is somewhat limited, as CD56 is not specific for NK cells; it is also expressed by T-cell subsets. However, our failure to detect CD56^+^ cells suggests that potentially pathogenic [16] NK cells are not present in GPA granulomas. We conclude that NK cells are unlikely to contribute to GPA granuloma formation or, alternatively, that they disappear during granuloma development. In any case, a potential pathogenic role of NK cells (e.g., by killing endothelial cells, as suggested by Tognarelli et al. [16]) in renal vasculitis whereby NK cells might also act via the luminal vessel side cannot be excluded. Beyond that, the different expression pattern of CD56 constitutes a clear difference between GPA granulomas and granulomas of classical granulomatous diseases such as tuberculosis and sarcoidosis. This different pattern has not been described before and might have an impact on granulomatous inflammation research aside from GPA. The numerous CD56^+^ cells in tuberculosis and sarcoidosis are most presumably a mixture of NK cells and CD56^+^ T-cells (Fig. 1b). Concordantly, other groups have reported that NK cells can be found in granulomas, such as in tuberculosis [33, 34]. Of note, Moins-Teisserenc et al. described a syndrome resembling therapy-refractory GPA with strong tissue infiltration by NK cells [35].

The reasons for a local NK cell deficiency in GPA granulomas are unclear, but they might include the specific inflammatory environment or chemotaxis. As NK cells were not detectable in active granulomatous GPA lesions, a disease- or at least a granuloma-promoting role of NK cells seems unlikely.

The second major finding of this study is the increase of peripheral blood NK cell proportions in inactive GPA. This finding is underlined by the negative correlation to BVAS and the positive correlation to the duration of remission. Importantly, this was not seen in our inactive CD cohort. As there is no perfect CD for GPA, we used a mixture of other systemic inflammatory diseases. In the literature, the authors of most studies of autoimmune diseases (systemic lupus erythematosus, rheumatoid arthritis, Sjögren’s syndrome, and others) have reported decreased NK cells in peripheral blood [30]. To our knowledge, no study to date has shown an increase of NK cells above normal HC levels in rheumatologic patients in remission, as we describe here in GPA. The NK cell increase therefore seems characteristic for GPA.

Interestingly, a rare hematologic disease with chronic NK cell lymphocytosis [lymphoproliferative disease of large granular lymphocytes (LDGL)] is associated with autoimmune syndromes, including arthritis and vasculitis. NK cell lymphocytosis in these patients results from an aberrant expansion of an immature and functionally deficient NK cell population [32]. Therefore, in the third part of this study, we further characterized NK cells in GPA in relation to its activity. In contrast to NK cells in LDGL, NK cells in inactive GPA appeared to be normal mature NK cells. This is suggested by a normal subset distribution according to the expression levels of CD56, by the equipment with functional NK cell receptors, and by a normal response to target cells, including preserved discrimination between targets overexpressing specific NK cell receptor ligands. We therefore conclude that NK cells in both active and inactive GPA are normally immune-competent. We found no signs of hyporesponsiveness as are seen in several rheumatologic diseases [30]. Tognarelli et al. reported elevated surface expression of CD107a on NK cells from patients with GPA as an indirect sign of in vivo activation. Regardless of this, we did not find increased surface expression of CD107a on GPA compared with HC NK cells in our degranulation assay negative controls without activation by target cells (not shown).

Possible reasons for increased NK cell proportions might include changes in NK cell maturation, development, proliferation, or survival. In a large study of 137 patients with GPA, researchers found elevated inflammatory serum proteins in active GPA and a significant reduction of many but not all of those markers during the disease course [36]. Therefore, a disturbed regulation of NK cells by cytokines and chemokines could play a central role in explaining altered NK cell levels. Interestingly, elevated blood levels of IL-15 and IL-18 were reported in both active and inactive GPA [36]. IL-15 and IL-18 have crucial roles in NK cell homeostasis [37, 38]. On the basis of recently published data, a disturbed control of NK cells by regulatory T cells needs to be considered [39–41]. In inactive GPA, proportions of regulatory T cells are increased but reportedly functionally deficient [42]. Alternatively, increased blood proportions could be affiliated with a different migration of NK cells. A single study showed an upregulation of chemokine (C-C motif) receptor type 5 on NK cells in active GPA [43]. Together, these studies [36, 42, 43] render the NK cell increase in inactive GPA feasible.

Different immunosuppressive regimen and steroid dosages might also shift lymphocyte proportions. On one hand, patients with active GPA usually receive stronger induction therapy, which was associated with lower NK cell counts in our study. On the other hand, we saw increasing NK cell proportions in three of four patients who were followed during (re)induction therapy (data not shown), indicating an increasing effect of strong immunosuppression on NK cell percentages. The less potent maintenance therapy in inactive GPA was indirectly associated with elevated NK cell counts, but long-term remission was associated with less maintenance therapy and even more NK cells. Concerning steroid dosages, patients with inactive GPA received steroid dosages similar to those of patients with inactive CD without elevation of NK cell proportions. Furthermore, despite significantly lower steroid dosages in inactive CD compared with active GPA, NK cell proportions did not differ between these groups. As these contradictory observations exclude a clear increasing or decreasing effect of immunosuppression on NK cell proportions, a direct effect is unlikely. In summary, elevation of NK cell proportions is a correlate of clinical amelioration and not of alterations of medication.

A permanent challenge in treating GPA is to interpret new and unspecific complaints of a patient in remission, such as fever or night sweats, that could also be unrelated to GPA. To date, biomarkers of disease activity that can reliably confirm or exclude GPA activity are missing. The availability of such biomarkers could objectify treatment decisions that are to date based largely on the physician’s experience. Therefore, all recent reviews on this topic emphasize the importance of the identification of new biomarkers in GPA [8, 29, 44, 45].

In the present study, NK cell proportions in PBL >18.5 % determined GPA inactivity with a specificity of 100 % and a sensitivity of 71 %. Importantly, the positive likelihood ratio was >8, indicating that NK cell proportions might be a useful activity biomarker [29]. Future prospective studies with larger patient cohorts should be done to confirm our data and tighten a suitable threshold of normal and elevated (possibly protective) NK cell proportions. Accordingly, in routine clinical practice, elevated NK cell proportions might help to exclude GPA activity.

A better understanding of GPA pathogenesis is needed to design new, target-specific therapies. Together with the results reported by Tognarelli et al. (tissue origin–dependent endothelial alteration by cytokines and killing by NK cells) [16], our third finding suggests that abnormally high killing of renal MECs by NK cells is more a result of changed endothelial cells than of altered GPA NK cells, as the latter do not degranulate differently from HC NK cells in the presence of target cells from epithelial, lymphatic, and non-renal endothelial origins [21].

It would therefore be tempting to speculate that a potential involvement of NK cells in GPA pathogenesis is determined not by NK cell functionality but by the expression of NK ligands in inflamed tissues. The expression of NK cell ligands has been shown in different inflamed GPA tissues, including granulomas [14, 15]; in the present study, we show that GPA NK cells can recognize ligand-bearing target cells from origins other than the endothelium (Fig. 4).

Importantly, Holmén et al. reported that circulating inflammatory endothelial cells express major histocompatibility complex class I polypeptide-related sequence A(MIC-A), a ligand for NKG2D, and are pathogenic in GPA [46]. These cells positively correlated with disease activity [46]. As Tognarelli et al. showed that inflammatory endothelial cells can be killed by NK cells, and as we show a negative correlation of NK cells with disease activity, one could speculate that NK cells can clear the circulation from pathogenic inflammatory endothelial cells and thus have a protective role in GPA. Beyond that, NK cells might possess a regulatory function by controlling other immune cells (e.g., activated CD4^+^ T-cells) [25].

To date, the mechanisms underlying increased blood NK cell proportions in inactive GPA and their possible pathophysiological role in vivo remain unknown and should be investigated in future studies.

## Conclusions

Our study increases knowledge about tissue and blood distribution of NK cells in GPA. NK cells are not detectable in GPA granulomas. High NK cell percentages in peripheral blood are associated with clinical suppression of disease activity and might possibly be used as a biomarker to exclude GPA activity.

## Abbreviations

7-AAD: 7-aminoactinomycin
ANCA: antineutrophil cytoplasmic antibody
AUC: area under the curve
B7-H6: member of the B7 family of immunoreceptors, ligand for NKp30
BVAS: Birmingham Vasculitis Activity Score
CD: control disease
DMARD: disease-modifying antirheumatic drug
ENT: ear, nose, and throat
FSC: forward scatter
GPA: granulomatosis with polyangiitis
HC: healthy control
HE: hematoxylin and eosin stain
IgG: immunoglobulin G
IL: interleukin
LDGL: lymphoproliferative disease of large granular lymphocytes
mAb: monoclonal antibody
MEC: microvascular endothelial cell
NK: natural killer
NKG2D: natural killer group 2, member D, activating natural killer cell receptor
NKp30/NKp46: natural killer cell p30/46-related protein, natural cytotoxicity receptors, activating natural killer cell receptors
NS: nervous system
PBL: peripheral blood lymphocyte
PBMC: peripheral blood mononuclear cell
PR3: anti-proteinase 3 antibodies
ROC: receiver operating characteristic
SD: standard deviation
SSC: sideward scatter
Tb: tuberculosis
VC: vector control
